# Citizen Science, a promising tool for detecting and monitoring outbreaks of the crown-of-thorns starfish *Acanthaster* spp.

**DOI:** 10.1038/s41598-019-57251-8

**Published:** 2020-01-14

**Authors:** Pascal Dumas, Sylvie Fiat, Amaury Durbano, Christophe Peignon, Gérard Mou-Tham, Jayven Ham, Sompert Gereva, Rocky Kaku, Olivier Chateau, Laurent Wantiez, Antoine De Ramon N’Yeurt, Mehdi Adjeroud

**Affiliations:** 1 IRD, UMR 9220 ENTROPIE, BP A5, Nouméa, New Caledonia; 2 Fisheries Department of Vanuatu, PMB 9045, Port-Vila, Vanuatu; 3 Laboratoire d’Excellence “Corail”, 58 avenue Paul Alduy, 66860 Perpignan, France; 4 Aquarium des Lagons de Nouméa, 61 promenade Roger Laroque, 98 800 Nouméa, New Caledonia; 5 Université de Nouvelle-Calédonie, BP R4, 98851 Nouméa, New Caledonia; 6 Pacific Centre for Environment and Sustainable Development, The University of the South Pacific, Suva, Fiji; 7 IRD, UMR 9220 ENTROPIE, UPVD 52 avenue Paul Alduy, 66860 Perpignan, France

**Keywords:** Conservation biology, Tropical ecology, Invasive species

## Abstract

Monitoring potentially devastating coral-eating crown-of-thorns starfish (COTS) populations at scales relevant to management is a challenging task. Here, we investigated a citizen science approach to detect COTS outbreaks and prioritize management responses. Between 2014 and 2018, 38 000 COTS were recorded through 641 online observation reports submitted across New Caledonia, Vanuatu and Fiji by private stakeholders (51%), NGOs (22%), business operator (11%), research/government agencies (16%). COTS were observed in multiple areas, including in remote/inhabited reefs where they had never been reported. A three-level classification was developed to discriminate risk areas and propose operational guidelines to streamline management actions. About two-thirds of reports had low abundances (<10 starfish sighted) and could be addressed with low priority. Verification surveys at 65 reef sites confirmed outbreaks in half of the cases, along with high peak densities (7 000 ind.ha^−1^). Combining professional and non-professional observers increased the detection range (+27%) and the number of COTS detections (+129%). Citizen reports were eventually followed by removal campaigns organized within diverse institutional frameworks. While citizen monitoring has intrinsic limitations, we advocate that it constitutes a complementary and promising approach to support the ongoing management efforts in all countries affected by COTS.

## Introduction

Outbreaks of pest species are increasingly stated as a major threat affecting all marine ecosystems, with unprecedented impacts across a range of compartments including habitats, native biodiversity or associated human activities^1–3^. While historically the attention was first drawn to invasive (alien) species, the concept was progressively expanded to encompass uncontrolled expansion of any species with detrimental ecological or economic effects, regardless of its origin -i.e. including native as well as non-native species^4^. This is the case of the coral-eating crown-of-thorns (COTS) *Acanthaster* spp., a large opportunistic starfish with boom-and-bust cycles which is now recognized as a potential pest across its natural distribution range^5^. Managing unpredictable, potentially devastating COTS population outbreaks is a rising issue across the Indo-Pacific where this voracious coral predator is historically a threat^6^. Along with tropical cyclones and coral bleaching caused by increased seawater temperature, COTS outbreaks are a leading cause of coral reef decline, with mortality of scleractinian corals eventually reaching up to 90% during extreme events^7,8^. Major alterations of coral reef ecosystems resulting from massive coral loss and subsequent cascading effects on coral-associated fish and invertebrate species are now of increasing conservation concern throughout COTS distribution range^9,10^.

The origin and ultimate mechanisms underlying COTS outbreaks are not yet fully understood, despite considerable research efforts^11^. While treating the symptom without fully addressing the causes certainly limits success, there is now an unequivocal need for pragmatical and effective management approaches to i) prepare for the occurrence and ii) contain the spread of future potential outbreaks^12^. As for most pest species, early and reliable detection of outbreaks before they become established is the first, critical step for success. However, this is a challenging task due to methodological and financial issues in repeatedly surveying large and/or remote reef areas, as well as to the absence of a universal, unequivocal criterion to discriminate ‘normal’ vs. ‘outbreak’ populations^13^. Long-term, government-led programs with standardized underwater census methods such as those implemented over the last decades in Guam, American Samoa, Hawaii, or the Great Barrier Reef constitute powerful tools to address both conceptual and practical aspects of COTS monitoring^14^. However, their cost may be well beyond the reach of most small Pacific island countries (PICs) which often lack scientific/technical expertise, dedicated human resources, and long-term financial support^15^.

In this study, we investigated for the first time the efficiency of a citizen science workflow to detect and document COTS outbreaks and prioritize management responses at large (country, region) scales. Citizen science, also referred to as public participation in scientific research, is increasingly advocated as a means to overcome data limitations when large scales are considered, and scientific resources are limited^16^. Here, we obtained georeferenced COTS reports from a wide array of marine stakeholders in three Pacific island countries affected by recurrent outbreaks. Citizen reports from New Caledonia, Vanuatu and Fiji were collected through a network of partner websites and mobile applications through a standardized API (application programming interface). This information was used to identify and map priority risk areas for COTS at country scale, raise awareness and inform decision-makers. Verification surveys using standardized underwater visual census (UVC) methods were then conducted by professional observers to assess the ability of citizen monitoring to detect COTS reliably, and to help the operational management of outbreaks.

## Results

### Contribution of citizen science monitoring

**Figure 1:**
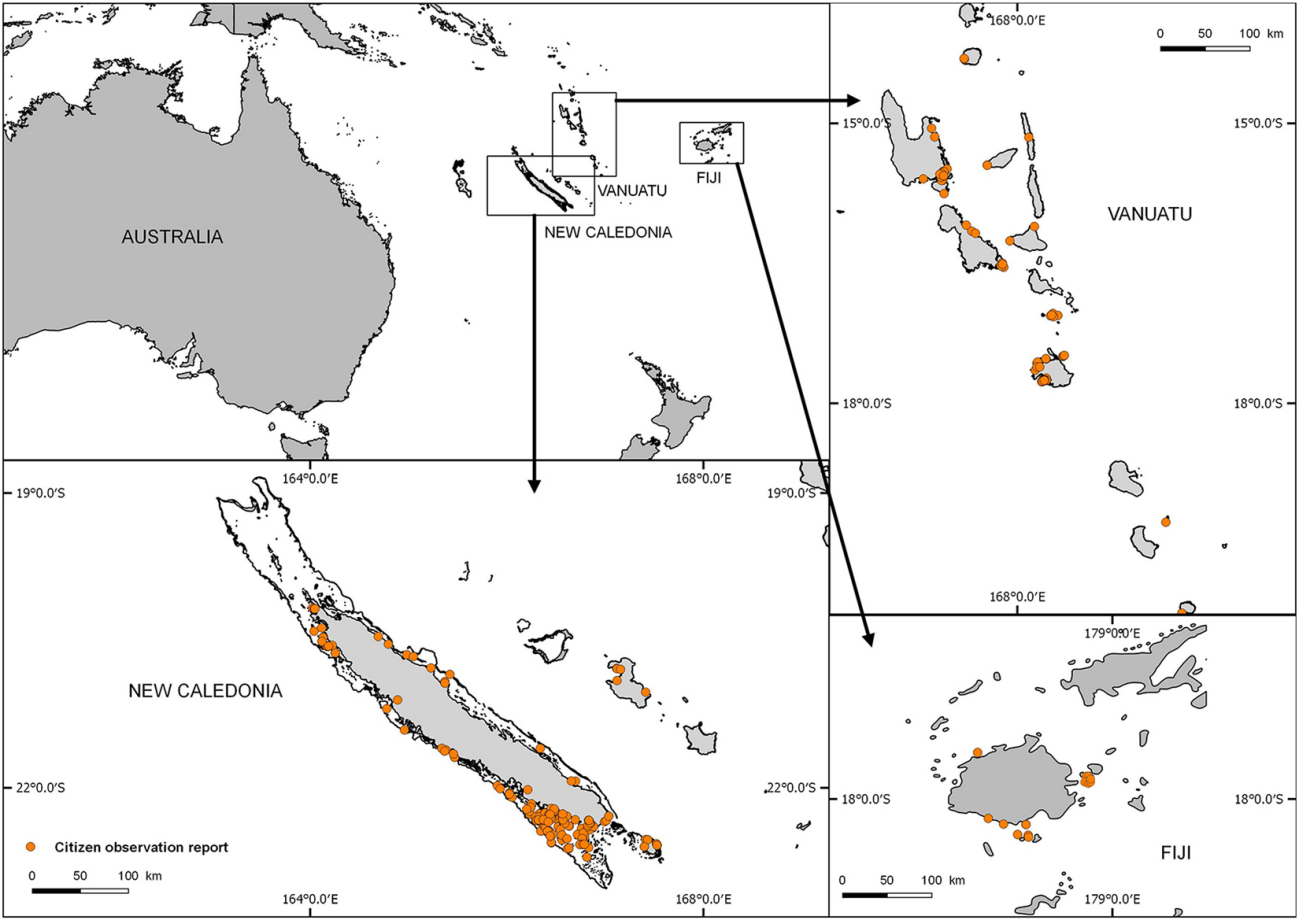
Spatial distribution of COTS participative reports collected during the OREANET project.

**Figure 2:**
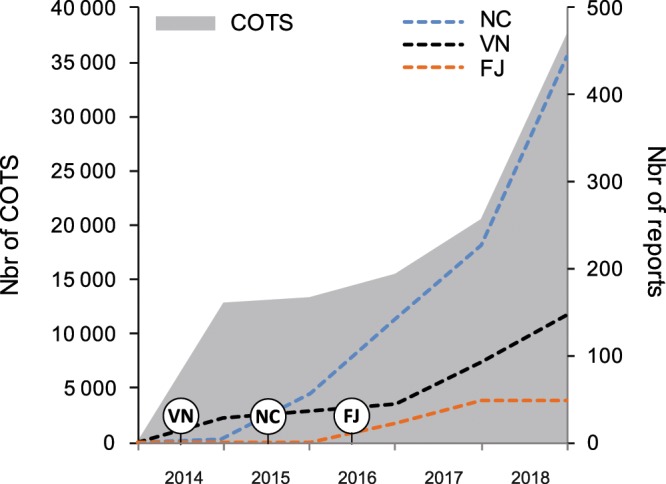
Evolution of citizen monitoring during the OREANET project. COTS cumulated abundances, number of collected reports with dates of project start in the three target countries. *Grey area: COTS abundance; dashed lines: COTS reports per country; VN: Vanuatu; NC: New Caledonia; FJ: Fiji*.

The number of monitoring reports submitted between 2014 and 2018 ranged from 46 to 271 per year, reaching a total of 641 COTS observation reports recorded over five years. Contributions mainly came from New Caledonia (445 reports) followed by Vanuatu (148 reports) and Fiji (48 reports). In these countries, the observations were not homogeneously distributed spatially. Most reports came from areas close to the main cities or touristic areas: the South West lagoon surrounding Nouméa city in New Caledonia, Efate and Santo islands in Vanuatu, and Viti Levu island in Fiji. A number of reports were sent from more isolated, remote and/or less populated areas such as Northern and Southern Vanuatu islands (Torba and Tafea provinces); the Eastern Loyalty islands Group in New Caledonia; the Central Lomaiviti Group in Fiji (Fig. 1). Altogether, about 38 000 COTS were reported by volunteer participants, the highest numbers being in Vanuatu (19 893) and New Caledonia (17 001) (Fig. 2). In the latter countries, the data were mostly collected through the online forms of the host entities websites, in particular the National Fisheries Department in Vanuatu (https://fisheries.gov.vu) and the French National Research Institute for Sustainable Development in New Caledonia (http://oreanet.ird.nc/). With less than 30 downloads from online stores between 2016 and 2018, the use of the official OREANET mobile applications to report on COTS was negligible (<5% of the reports). However, a partnership developed in 2018 with a local service company resulted in a marked increase, with 14% of the 2018 reports sent through the MAREES NC (http://www.cocogeek.nc/) mobile application in New Caledonia.

**Figure 3:**
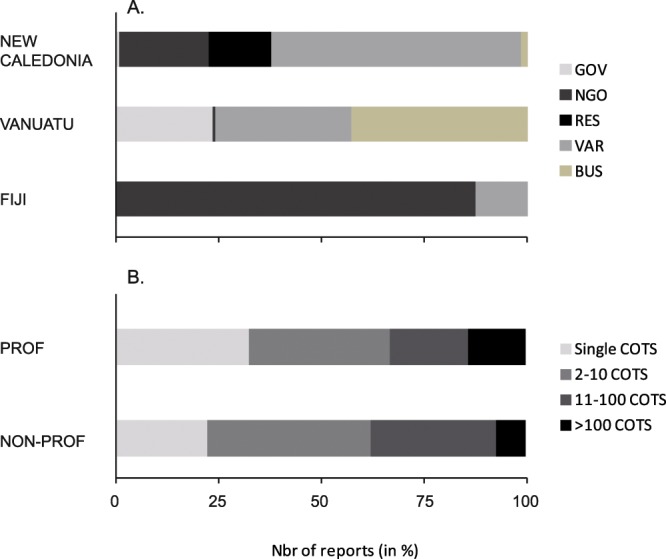
Typology of the participative reports collected during the OREANET project. (**A**) Stakeholder categories per country and. (**B**) COTS abundance patterns for professional vs. non-professional observers. *PROF: professional observers; NON-PROF: non-professional observers; GOV: government agencies; NGO: non-governmental organizations; RES: research organizations; BUS: small business operators; VAR: others stakeholders including private individuals such as recreational snorkelers, divers, fishermen etc*.

Overall, reports were mostly submitted by private individuals (including recreational snorkelers or divers, artisanal or subsistence fishers; 50.9%), followed by NGOs (21.7%), research organizations (10.5%), small business operators (10.8%) and government agencies (6.2%). This ratio varied among countries. Along with a major contribution from recreational snorkelers, divers and fishers (60.9%), New Caledonia had significant contributions from both research organizations and NGOs (15–20% each) and almost none (<2%) from government bodies and small business operators. A different situation was found in Vanuatu, where most COTS reports originated from local business operators (42.6%) and private individuals (snorkelers, divers and fishers, 33.1%). Government departments were also involved in reporting (23.6%), while NGOs or research institutes had very limited contributions in Vanuatu (<1%). In contrast, the relatively few reports from Fiji were mainly submitted by NGOs (87.5%), and very little by private individuals (12.5%) (Fig. 3a).

### Observers’ contributions to COTS detection

In total, observers’ reports of low COTS numbers (≤10 COTS per report) were most frequent (63.4% of the records), including single, isolated specimens (25.1%) spotted on the reefs by snorkelers or divers and low aggregations (38.3%). Moderate COTS aggregations were suspected on a regular basis (>10 specimens in a single report, 27.2%), with a few observations of large aggregations in each country suggesting potential outbreaks (>100 COTS in a single report, 9.4%). The abundance patterns detected by volunteer vs. professional observers exhibited slight differences (Chi Square test, n = 638, p < 0.001): single specimens and large aggregations were more frequently reported by professional observers, while non professionals comparatively detected more ‘intermediate’ situations (low and intermediate aggregations) (Fig. 3b).

**Figure 4:**
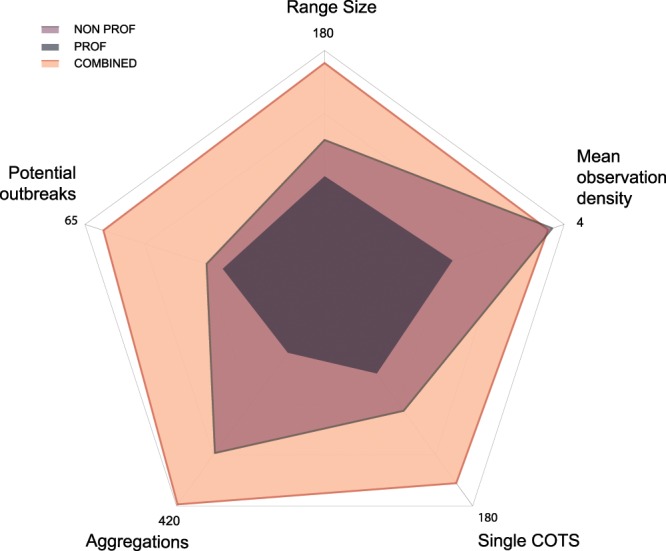
Contribution of professional vs. non-professional observers to the detection of COTS. Spatial range of COTS occurrences, mean observation density and number of COTS detection per abundance categories for professional (PROF), non-professional (NON PROF) and combined observers. *Range size: number of 5 km* × *5 km geographical quadrats that COTS were detected in; Mean observation density: average number of COTS reports per geographical quadrat; Single COTS: number of reports of single COTS; Aggregations: number of reports with less than 100 COTS; Potential outbreaks: number of reports with more than 100 COTS*.

The information provided by professional and non-professional observers was complementary; in fact, the combined datasets increased the spatial range of COTS occurrence (i.e. the geographical areas were COTS were detected) and the number of COTS detection within the range limits. Volunteers had the highest contribution and submitted more observation reports than professional observers (+129%), whatever the abundance categories (single COTS, aggregations, potential outbreaks). They also covered larger areas and detected COTS in 116 geographical quadrats, i.e. a 27% increase relative to professional observers (91 quadrats) at study scale (Fig. 4). Overall, the number of reports per area was similar for both observer categories (3.8 reports per geographical quadrat for volunteers vs. 2.16 for professionals, Mann-Whitney U test, n = 207, N.S.).

### Scientific surveys

**Figure 5:**
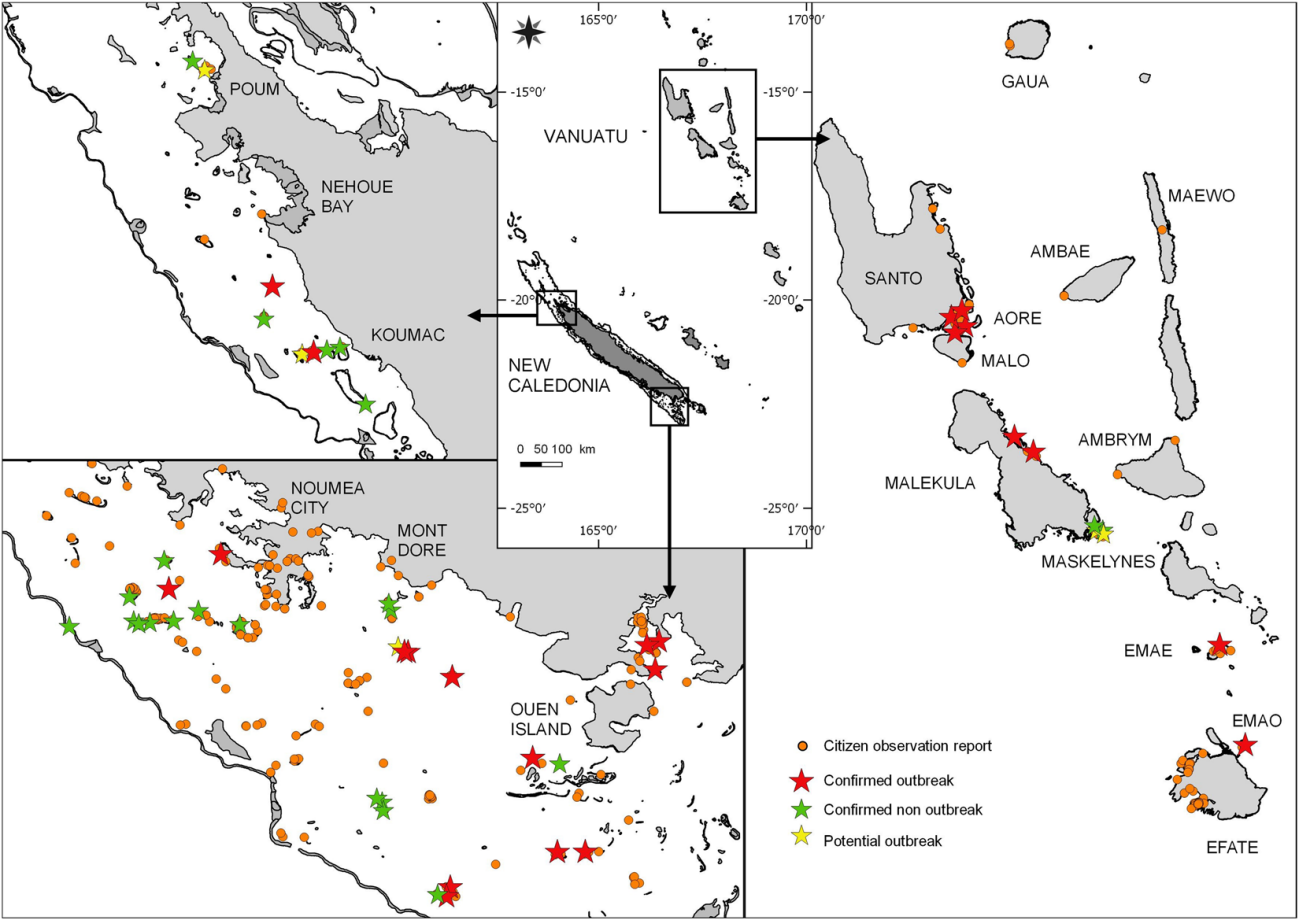
Spatial distribution of COTS populations in Vanuatu and New Caledonia during the OREANET project. Location and observed demographic status of natural populations of *Acanthaster solaris* in geographical areas where potential outbreaks were reported.

**Figure 6:**
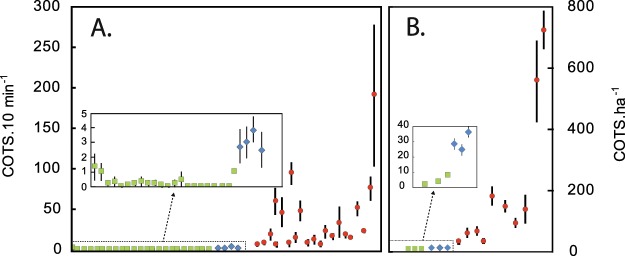
Distribution of COTS abundances in A. New Caledonia and B. Vanuatu. Results of UVC scientific surveys conducted across the 65 verification reef sites where citizen reports suggested potential outbreak populations of *Acanthaster solaris*. Means ± SE for standardized 10 minutes-swims and belt transects.

In total, 15 389 COTS were individually counted during 2 468 scientific surveys (1 556 belt transects, 912 timed swims) across our 65 verification sites. The presence of COTS starfish was confirmed in most (87.7%) of the sites, generally with elevated abundance levels (mean 12.4 ± 42.3 ind. per swim/224 ± 597 ind.ha^−1^ for timed swim and belt transect surveys, respectively). Based upon our abundance/density thresholds, the occurrence of an outbreak was confirmed in 47.7% of the surveyed sites and excluded in 40.0%; 12.3% of the sites had intermediate abundance values and were classified as potentially affected, thus requiring further information (Fig. 5). Sites with confirmed outbreaks were found in both New Caledonia and Vanuatu; outbreak populations exhibited contrasted abundance levels across sites, with means ranging from 35 to more than 722 ind.ha^−1^/6 to 190 ind. per ten-minute swim (Fig. 6). Extreme values were occasionally observed when surveys coincided spatially with dense COTS aggregations (maximum of 605 ind. per ten-minute swim and 7000 ind.ha^−1^) (see Supplementary Table S1).

## Discussion

Citizen science plays a major role in most regional coral reef programs, where monitoring initiatives increasingly rely on volunteers to collect information on a growing array of species, including invasive pests^17–19^. While the COTS starfish *Acanthaster solaris* is not an introduced species in the Pacific, it has all the ecological characteristics of a pest (e.g. extreme fecundity, population cycles with unpredictable high-amplitude, large-scale density fluctuations, opportunistic feeding behaviour, and significant impacts on other ecosystem components, including human activities). As a consequence, it was recently proposed to apply similar management approaches typically used for invasive species to COTS^5^. Effective detection and reliable monitoring are core characteristics of pest management frameworks^20^. For COTS, large (Pacific-wide) geographic distribution, fast propagation and potentially high recurrence of outbreaks create multiple monitoring challenges; in fact, most Pacific island countries may lack the resources to document COTS patterns at scales relevant to develop long-term efficient management strategies. This is a typical case where citizen monitoring has the potential to produce data with sufficient spatial coverage and temporal replication which would not otherwise be affordable. In the Pacific, COTS are often targeted opportunistically in participatory monitoring initiatives (led by national/provincial authorities, e.g. the ‘Eye on the Reef’ program on the GBR, or community-based^21,22^); however, to our knowledge, this is the first time a COTS-dedicated citizen science approach was planned and implemented on a large (country/regional) scale.

Overall, this study clearly demonstrated the potential of citizen science to provide relevant information on COTS distributions on an operational scale. Volunteers were able to confirm the presence of COTS and potential population outbreaks in multiple reefs across New Caledonia and Vanuatu, including in remote and/or inhabited areas where they had never before been reported. This information is particularly valuable in PICs, where baseline information on COTS distribution is usually lacking, and geographical fragmentation entails substantive issues to monitor distant reef systems. This is the case in Vanuatu, where quantitative data on COTS distribution across the ~82 islands (65 of them inhabited) is very scarce despite informal reports by local communities and business operators during recent decades^21,23,24^. COTS aggregations were first documented across the archipelago in 1989–1990^25^. Successive waves of starfish were then reported across the northern province since 2004, then in the central province since 2006 (S. Gereva, pers. comm.). More than ten years later, our results confirm that dense starfish populations are still occasionally occurring in these areas, with an outbreak confirmed in at least five reef sites located in the southern Santo area and two sites in Efate between 2014 and 2018. Outbreaks were also detected and confirmed in previously undocumented areas such as Malekula and Emae islands, along with timely observations of starfish aggregations in all six Vanuatu provinces. Our results revealed that COTS are in fact widely distributed across the entire Vanuatu archipelago, with densities eventually reaching peak values similar to, or even higher than, the highest densities commonly reported from the literature (e.g. peak density of 7000 ind.ha^−1^ recorded in Ratua and Aore islands, Sanma province). In New Caledonia, high densities of COTS have only been formally reported on some occasions during the last decades. In 1983, densities of up to 3.3 COTS per 100 m^2^ were recorded at ilot Maître, a mid-shelf reef situated 2 km south-west of Nouméa city^26^. Similar events were reported in 2000 in the same area, then again in 2012 across the South West lagoon of New Caledonia where subsequent scientific surveys revealed the highly localized and ephemeral character of COTS outbreaks^27^. With 445 observations sent by stakeholders and 23 confirmed outbreak sites over the course of the study, our results further confirm that COTS constitute an overlooked issue in New Caledonia. In addition to dense aggregations (e.g. peak density of 10 800 ind.ha^−1^ recorded at To islet in February 2018^28^), they also emphasize that the spread of COTS and therefore their impacts may be substantially wider than previously expected. This is especially the case in the less populated northern and eastern lagoon areas or in the offshore Loyalty Islands, where citizen reports are scarce. In Fiji, historical reports indicate that COTS were relatively common between the 1920s and 1960s, with some well-remembered outbreaks in the 1920s and 1940s^29^. The latter authors carried out a 13-year COTS recruitment survey between 1976 and 1989, which showed a peak in spawning during the hottest period of each year. Since 1990 there has been virtually no dedicated study on COTS in Fiji, but data suggest an escalating frequency and severity of outbreaks affecting certain locations^30^. This work provides evidence that COTS still constitute a potential threat in this highly fragmented archipelago.

Our results further emphasize that citizen reports may help inform managers and decision-makers to select priority risk areas. Both professional and volunteer observers tended to report frequently on aggregations. However, about two-thirds (63.4%) of observations had low abundances (fewer than ten starfish sighted) and could typically be addressed with low priority if monitoring resources were limited. From the remaining reports with middle to high starfish abundances, scientific surveys confirmed the occurrence of outbreaks in about half (47.7%) of cases. Of course these numbers should be taken with care, as ultimately all field surveys are proxies. The standardized verification surveys used in this study were in particular conducted on snorkel rather than SCUBA. While this significantly increased our sampling effort (e.g. in remote areas in Vanuatu where SCUBA entails major logistical constraints), it also restricted detection to shallow reef areas (maximum 5–6 m). This may punctually result in under-reporting of potentially dense populations ascending from deeper areas, especially cryptic juveniles (up to 2 years old) which may settle at the base of reef slopes, and then gradually move upwards as they grow and mature^31,32^. Ideally, combining different verification methods would increase our efficiency to detect increasing or outbreak densities, a prerequisite for timely intervention^33^. However, and keeping in mind our still limited understanding of COTS outbreak initiation and the current limitations of scientific surveys, these findings constitute operational guidelines to streamline management actions when outbreaks are suspected. This was for instance the case in Vua, an inhabited patch reef located 45 km south-east of Nouméa (ID: NC67, cf. Supplementary Table S1). In this remote area characterized by little human activity except recreational fishing, the emergence of COTS was reported by a snorkeler in November 2015 and the site was immediately ranked a high priority. Subsequent verification surveys confirmed the spread of a severe outbreak over the following months, with peak density reaching 5200 ind.ha^−1^. This eventually led the provincial authorities to authorize a participative removal campaign as a trial run, where more than one ton of COTS (1377 individuals) were treated by lethal injections of vinegar following the method of Moutardier *et al*.^34^. This management strategy was expanded in 2018 by a provincial ordinance, and culling campaigns were authorized in four additional sites where citizen data reported COTS outbreaks (South West lagoon, NC517, NC518, NC519, NC549).

A rapid decision process is crucial to target effective management responses, as the progression of pest outbreaks can be very fast. In New Caledonia, verification surveys following a volunteer report emphasized a dramatic increase of COTS between June and October 2017 in the previously unaffected Uimé reef (ID: NC299; peak density 7 000 ind.ha^−1^), followed by massive coral devastation and the complete disappearance of starfish populations by February 2018. Similar observations were made in Vanuatu, where a severe outbreak quickly spread across the fringing reefs of the Crab Bay marine protected area during 2014^35^ (ID: VN26; peak density 4050 ind.ha^−1^). Managing such highly ephemeral yet potentially destructive COTS outbreaks constitutes a considerable challenge. Along with a limited window of opportunity to detect COTS increases before they disappear, the potential lag between citizen reports and subsequent field validation entails risks that some outbreaks remain unrecorded, and some reports misinterpreted (‘false negative’). This may have occurred in some of our sites classified as unaffected or potentially affected, as meteorological and logistical issues resulted in verification delays eventually reaching several weeks, and a maximum of 14 months in one of the most remote areas. Shortening the verification times for suspicious reports is key to prevent unnoticed transitions to outbreak densities.

Our results further suggest that management outcomes will largely depend on the flexibility of local governance and the capacity of decision-makers to adapt their strategies to rapidly evolving situations. In PICs, management of coastal ecosystems is typically undertaken within multiple institutional frameworks —from traditional village authorities such as chiefs, community leaders, customary landowners to provincial, regional, and central governments. Each system has different strengths and weaknesses which will affect their responsiveness to address highly unpredictable, rapidly moving, and ephemeral outbreaks. Flexible responses may be more likely when governance is straightforward and greater management responsibility is given to communities. In Vanuatu, COTS removals are often decided ‘on the fly’ and implemented at village scale by the traditional authorities, in particular chiefs and chief’s councils^36^. For instance, community-based cleanups supervised by qualified officers of the Vanuatu Fisheries department could be organized through minimal administration effort following COTS reports in Emao, Aore and Emae islands (VAN 10, VAN 16 & VAN21). In other areas such as in the main island of Efate, successful arrangements were made to allow local NGOs and/or small business operators to conduct COTS monitoring and removal campaigns, with minimal supervision by the central government. In contrast, about 18 months were eventually necessary to authorize removal campaigns in New Caledonia, whose centralized marine regulation framework does not recognize COTS as a pest. Lightening the administrative procedures to i) declare an outbreak following citizen observation and ii) authorize control campaigns will be required to take full advantage of citizen science data, and to develop a more integrated management strategy. Of course long-term commitment may be more achievable when COTS management is specifically included in national or regional policies and specific funding is sourced - e.g., on the GBR, where a total of $2.4 million was eventually committed to COTS control programs by the Australian government^37^. However, this is not the case in most Pacific countries, such as in Fiji, Vanuatu or New Caledonia where the lack of recognition at higher decision levels impedes their ability to efficiently address the COTS issue in the long term, and most initiatives may be project-based.

Citizen monitoring was found to be very promising given the congruence between data collection, ecological patterns, and management scales. Our results in particular suggest that beyond their numbers, volunteers tend to opportunistically detect COTS in remote or isolated reef areas that may not be covered by professional observers - such as scientists, NGOs, government observers etc. who are often working in spatially-defined, project-based areas -, hence significantly increasing the detection range and the probability of early detecting abnormal or outbreak populations. However, it has intrinsic limitations. Correctly identifying the target species to document biodiversity can be challenging for non-specialists, especially in the context of multi-species surveys. This was not an issue here, as morphological identification is usually not considered a significant source of bias for COTS -at least at the genus level (*Acanthaster* spp.), which has very distinctive features that prevent confusion with other starfish. However, errors in identification are likely to occur eventually in certain socio-environmental contexts, e.g. where environmental education is weak, people have limited access to educational resources, or in areas which have not been recently affected by COTS. This was experienced once in a remote community in Vanuatu, where alleged COTS were found to be sea urchins of the *Diadema* genus. Personal commitment and therefore networking are also key factors in determining the success of citizen science approaches, which are usually fostered by the idea of collaborating and sharing knowledge rather than by direct financial pledges^38^. Most of the OREANET observation reports (69%) were submitted by non-professional data collectors, including members of coastal communities, recreational fishers, snorkelers, divers, boaters, dive operators etc. whose primary drivers were environmental and/or conservation concerns. Consequently, the geographical coverage of observation reports was highly heterogeneous, with major gaps in remote and/or low populated areas, in areas with limited access to communication (internet) networks and mobile technologies, or in areas where people may exhibit lower commitment to the protection of their reefs. Efficient, sustained networking with the local institutions and stakeholders was therefore of major importance. This may for example explain the discrepancy observed between Fiji (where reporting was minimal) and New Caledonia/Vanuatu (which both totalize more than 90% of the citizen reports). While the latter countries benefited each from a local facilitator in charge of networking aspects, in Fiji logistical constraints prevented the recruitment of a dedicated coordinator. As a result the project was managed remotely, and the connection with the local partners was much weaker.

Another limitation is that starfish counts do not constitute absolute or universal indicators of outbreaks, which can be defined based upon various ecological or methodological perspectives^31,39^. Sustainable density, above which coral assemblages are negatively affected by COTS, is of particular importance for managers; however, it will vary locally depending upon COTS population and coral reef characteristics and trajectory^12^. Moreover, marked variations in background densities seem to be a natural feature of COTS dynamics^14^, hence challenging the use of invariant abundance/density thresholds. The numbers of COTS reported by non professional observers also reflected contrasted survey strategies and observation methods, as a compromise between the managers’ needs for standardized, reliable data and those of the volunteers for a flexible, rapid reporting process. From our results, typical non-professional citizen reports are collected during 30–60 minute swims covering everyday activities (snorkeling, spear fishing, scuba-diving etc.), and do not specifically focus on COTS. In contrast, reports sent by professional observers may derive, at least partly, from monitoring projects specifically targeting COTS. This may hinder comparisons, as different sampling techniques have different bias which impact the detectability of COTS and may require specific thresholds levels to define an outbreak^13,40^. Significant differences are for example usually observed between day and night surveys, such as in Moorea (French Polynesia) where Kayal *et al*. (2017) recorded an average increase of 27% in COTS abundances at night during a massive outbreak^41^. While from our experience most citizen observations were made during the day, some night reports may have been sent by recreational divers and snorkelers, or by fishermen in areas/countries where night fishing was not prohibited. Other environmental and/or biological factors - including reef topography and structural complexity, water visibility and depth, meteorological conditions, COTS sizes and density - may also affect the detectability of COTS populations, especially during the initiation phase of an outbreak when local densities are well below threshold densities^41–43^. Care should therefore be exercised in interpreting the records: while the decision process is quite straightforward when confronted with values towards the upper or lower ends of the range, intermediate densities will likely require additional monitoring effort to elucidate potential outbreak situations. From a more pragmatic perspective, starfish counts should also be balanced with their spatial distribution, as highly aggregative behavior can result in very patchy patterns where only certain parts of the reefs will have abnormal densities. This was demonstrated in Aniwa island (Vanuatu) in November 2014, where COTS were observed in high numbers across a 50m-long section of a previously unaffected fringing reef system, but were absent from the 77 randomly-laid belt transects.

«Large-scale environmental science requires citizen science»^44^. With the growing popularity of participatory approaches, non-experts are ultimately considered a highly valuable support to research. This is particularly relevant for studies on COTS and similar pest outbreaks, as citizen monitoring may be one of the few practical ways to cope with the ecological scales required. The citizen science workflow described in this paper potentially allows to expand the detection of outbreaks in areas where baseline information is usually lacking, e.g. in small island countries where geographical fragmentation entails substantive monitoring issues. There are obvious, intrinsic limitations to this approach, such as the trade-offs between data quality and quantity, the dependence upon personal commitment, and the potential gaps between scientific expectations and achievable outputs. In fact, further academic research will be required to develop a better (holistic) understanding of outbreaks, a prerequisite to address more operational aspects of “the acanthaster phenomenon” (sensu Moran^6^). However, keeping in mind that citizen monitoring will not replace scientific surveys, we advocate that it constitutes very promising approach to support and improve the ongoing monitoring and management efforts in all the countries affected by COTS.

## Methods

### The OREANET (oceania regional acanthaster network) project

#### Data collection tools

Customized tools were developed to ensure a quick and reliable data collection by all partners. We distinguished two types of partners: i) the host entities, which were responsible for collecting, centralizing and hosting the data in each of the network’ target countries, and ii) the implementing entities, which could be private or public groups facilitating the collection of citizen data through their own networks, websites or mobile applications. We developed a standardized online COTS report form made as a CMS (Content Management System) plugin that can be easily integrated into the websites of the host entities. The citizen observation data were saved in a local database together with the website of the host entity, with export facilities available for COTS managers. A hybrid offline-capable mobile application was also created, to eventually counterbalance low internet accessibility in the region. It used a dedicated API allowing the application to store data in offline mode. The API was secured, so that implementing entities could reliably send their reports using identification keys. As a result, a website plugin and a mobile application were deployed in close succession from 2014 to 2016 in Vanuatu, New Caledonia, and Fiji, respectively.

#### Standardized reporting

The core of the reporting process was a standardized, user-friendly online report form, which included an interactive map and a free geocoding API for geo-referencing the citizen observations. It was composed of twelve fields referring to observer, observation context, and COTS. Mandatory information included observer name and contact details, date, location and context (snorkeling/SCUBA diving, depth range), and the number of COTS observed. We did not impose specific survey methods for citizen observers, in order to collect opportunistic reports during a broad range of marine activities. However observers were asked about the duration of the swims and the distance covered. To simplify the spatialization process, the observations were georeferenced by clicking the corresponding reef areas on a satellite map. The COTS abundances could be entered as quantitative, semi-quantitative, or qualitative (e.g., “10”, “dozens”, “large aggregations”, etc.) to suit highly diverse users’ profiles. More advanced users were encouraged to provide additional information such as specific details on COTS populations (e.g. detection of small, i.e. juvenile individuals), survey methods (e.g. duration of timed swims, dimension of belt transects) and the reef environmental context (e.g. estimates of live coral cover, presence of white feeding scars characteristics of recent predation by COTS, etc.). Once send, the observation reports were validated by a project moderator before being displayed online and made accessible to the public.

### Validation of volunteer data

We carried out quantitative COTS verification surveys in areas where citizen reports suggested the occurrence of outbreaks, using standardized UVC methods. Sites were primarily selected based upon the abundance levels reported, with sites with medium to high abundances (>10 COTS in at least a single report) preferred over those with observations of single specimens. In total, we surveyed COTS at 65 reef sites spanning seven degrees of latitude between Southern New Caledonia (22.6°S) and central Vanuatu (15.5°S). At each site, data were collected by a team of two to five snorkelers swimming parallel to the reef edge during the day. Whenever possible, COTS densities were estimated using 100 m^2^ belt transects; when meteorological or logistical conditions made the use of transects impracticable, COTS abundances were recorded using standardized ten-minute swims^45–47^. For all the surveys, the considered depth range was 1–5 m, with 5–10 m between observers to avoid overlap. The position of the timed swim or belt transects was recorded by a handheld Garmin^TM^ Map60Cx GPS device in an underwater housing.

### Analyses

#### Citizen data

For each country, we examined the numbers of reports and starfish reported, along with the contribution of the different observer categories to the reporting. The citizen reports were classified by the observer’s levels of expertise: professional (i.e. scientists, experts, trained volunteers etc.) vs. non-professional observers; and by broad socio-professional categories: private individuals (e.g. recreational fishers, snorkelers/divers, members of coastal communities etc.); small business operators (e.g. tourism operators, dive operators); NGOs; local government bodies; research organizations. COTS abundances were further classified into semi-quantitative abundance categories: single starfish; low aggregations (2–10 starfish); intermediate aggregations (11–100 starfish), and potential outbreaks (>100 starfish per observation). We compared the distributions of COTS abundance reported by professional vs. non-professional observers using a Chi-Square test.

Finally, we examined the extent to which the contributions of professional and non-professional observers increased the occurrence and the spatial range of COTS detection. First, the spatial distribution of COTS occurrences was mapped across the three project countries. We then constructed equal geographical quadrats (5 km × 5 km) covering the reef and lagoon areas; in each quadrat, we estimated the occurrence of COTS (presence or absence) and the mean number of COTS records. The number of reports sent by professional vs. non-professional observers were compared using a Mann-Whitney U test. All analyses were performed using QGIS 3.2 and Statistica v10.

#### Scientific data

In the literature, density thresholds indicative of outbreak populations usually fall within two main groups: values of around 100–150 ind.ha^−1^ and values of 10–15 ind.ha^−1^ (review in Pratchett *et al*.^12^). We first used these values to define a conservative, three-level density-based classification to distinguish between low (non-outbreak), medium (potential outbreak), and high (outbreak) COTS populations. For timed-swims where observers do not have a visual reference, we used an empirical conversion ratio based upon the average reef surface covered during a typical ten-minute swim to derive thresholds expressed in numbers of COTS per timed swim. The area surveyed was calculated by using the linear distance travelled along the reef edge multiplied by an estimate of the “path width” where observers actually look for COTS. While this value actually varies as a function of depth, reef complexity, visibility and observer etc., we used an empirical value of 2 m (Table 1).

**Table 1:** Abundance/density thresholds used to classify the COTS populations during scientific surveys.

Classification Level	Density (COTS.ha^−1^)	Abundance equiv. (COTS.10 min^−1^)
Non outbreak	<15^a^	0–1
Potential outbreak	15–100^b^	2–5
Confirmed outbreak	>100^c^	>5

^a^Endean 1974; Keesing 1990; Clark & Weitzman 2006.

^b^Dana *et al*. 1972; Keesing and Lucas 1992; Moran and De’ath 1992.

^c^Pearson and Endean 1969; Glynn 1973; Endean and Stablum 1975.

## Supplementary information

Supplementary Table S1: Summary of COTS population data in the 65 verification sites.
