# Supplementary material for: Citizen Science, a promising tool for detecting and monitoring outbreaks of the crown-of-thorns starfish Acanthaster spp
Source: Sci Rep. 2020 Jan 14;10:291. doi: 10.1038/s41598-019-57251-8 (PMC6959260; doi:10.1038/s41598-019-57251-8)
Supplement: Supplementary file 1 — Supplementary Table S1: Summary of COTS population data in the 65 verification sites. [file 41598_2019_57251_MOESM1_ESM.docx]

**Supplementary information**

**Citizen Science, a promising tool for detecting and monitoring outbreaks of the crown-of-thorns starfish *Acanthaster* spp.**

*Pascal Dumas, Sylvie Fiat, Amaury Durbano, Christophe Peignon, Gérard Mou-Tham, Jayven Ham, Sompert Gereva, Rocky Kaku, Olivier Chateau, Laurent Wantiez, Antoine De Ramon N’Yeurt & Mehdi Adjeroud.*

**Supplementary Table S1: Summary of COTS population data in the 65 verification sites.**

Total number of observation units, total number of COTS and abundance/density values per site from expert-based surveys. Mean, SE and range expressed in COTS per ha or COTS per swim for belt transect (TR) and timed-swim (TD) surveys, respectively. Population status: 1.Low (no outbreak); 2.Medium (potential outbreak); 3.High (confirmed outbreak).

| SITE ID | METHOD | NB.OBS. | COTS TOTAL | MEAN |  | SE | RANGE | STATUS |
| --- | --- | --- | --- | --- | --- | --- | --- | --- |
| NC217 | TS | 3 | 4 | 1.3 | per 10 min | 0.7 | 0 - 3 | 1 |
| NC238 | TS | 12 | 33 | 2.8 | per 10 min | 1.1 | 0 - 12 | 2 |
| NC239 | TS | 3 | 3 | 1.0 | per 10 min | 0.5 | 0 - 2 | 1 |
| NC242 | TS | 10 | 30 | 3.0 | per 10 min | 1.0 | 0 - 9 | 2 |
| NC245 | TS | 16 | 40 | 2.5 | per 10 min | 1.1 | 0 - 18 | 2 |
| NC246 | TS | 12 | 81 | 6.8 | per 10 min | 2.0 | 0 - 21 | 3 |
| NC247 | TS | 24 | 5 | 0.2 | per 10 min | 0.1 | 0 - 3 | 1 |
| NC248 | TS | 10 | 3 | 0.3 | per 10 min | 0.3 | 0 - 3 | 1 |
| NC249 | TS | 24 | 1 | 0.0 | per 10 min | 0.0 | 0 - 1 | 1 |
| NC262 | TS | 24 | 3 | 0.1 | per 10 min | 0.1 | 0 - 1 | 1 |
| NC263 | TS | 20 | 4 | 0.2 | per 10 min | 0.1 | 0 - 2 | 1 |
| NC264 | TS | 33 | 128 | 3.9 | per 10 min | 0.8 | 0 - 22 | 2 |
| NC266 | TS | 3 | 1 | 0.3 | per 10 min | 0.3 | 0 - 1 | 1 |
| NC267 | TS | 12 | 3 | 0.3 | per 10 min | 0.2 | 0 - 2 | 1 |
| NC268 | TS | 11 | 3 | 0.3 | per 10 min | 0.1 | 0 - 1 | 1 |
| NC294 | TS | 12 | 1 | 0.1 | per 10 min | 0.1 | 0 - 1 | 1 |
| NC295 | TS | 18 | 1 | 0.1 | per 10 min | 0.1 | 0 - 1 | 1 |
| NC296 | TS | 9 | 2 | 0.2 | per 10 min | 0.2 | 0 - 2 | 1 |
| NC299 | TS | 18 | 1074 | 59.7 | per 10 min | 15.2 | 0 - 214 | 3 |
| NC300 | TS | 6 | 205 | 34.2 | per 10 min | 16.6 | 6 - 122 | 3 |
| NC301 | TS | 2 | 1 | 0.5 | per 10 min | 0.4 | 0 - 1 | 1 |
| NC308 | TS | 6 | 288 | 48.0 | per 10 min | 10.7 | 1 - 80 | 3 |
| NC489 | TS | 6 | 97 | 16.2 | per 10 min | 2.9 | 5 - 26 | 3 |
| NC490 | TS | 10 | 506 | 50.6 | per 10 min | 7.6 | 17 - 95 | 3 |
| NC517 | TS | 9 | 200 | 22.2 | per 10 min | 6.8 | 2 - 62 | 3 |
| NC518 | TS | 10 | 754 | 75.4 | per 10 min | 13.3 | 27 - 153 | 3 |
| NC519 | TS | 39 | 3668 | 94.1 | per 10 min | 12.7 | 0 - 280 | 3 |
| NC549 | TS | 8 | 1523 | 190.4 | per 10 min | 81.0 | 28 - 605 | 3 |
| NC62 | TS | 9 | 79 | 8.8 | per 10 min | 2.0 | 0 - 17 | 3 |
| NC62b | TS | 4 | 34 | 8.5 | per 10 min | 2.9 | 1 - 16 | 3 |
| NC63c | TS | 16 | 300 | 18.8 | per 10 min | 6.2 | 0 - 82 | 3 |
| NC63d | TS | 2 | 31 | 15.5 | per 10 min | 0.4 | 15 - 16 | 3 |
| NC64 | TS | 4 | 72 | 18.0 | per 10 min | 2.5 | 10 - 24 | 3 |
| NC64b | TS | 2 | 2 | 1.0 | per 10 min | 0.0 | 1 - 1 | 1 |
| NC65 | TS | 8 | 183 | 22.9 | per 10 min | 1.3 | 20 - 30 | 3 |
| NC65b | TS | 8 | 0 | 0.0 | per 10 min | 0.0 | 0 - 0 | 1 |
| NC66 | TS | 20 | 127 | 6.4 | per 10 min | 2.5 | 0 - 42 | 3 |
| NC67 | TS | 8 | 99 | 12.4 | per 10 min | 4.6 | 1 - 43 | 3 |
| NC67b | TS | 16 | 742 | 46.4 | per 10 min | 16.9 | 0 - 210 | 3 |
| NC67c | TS | 50 | 370 | 7.4 | per 10 min | 3.1 | 0 - 134 | 3 |
| NC67d | TS | 20 | 303 | 15.2 | per 10 min | 5.6 | 0 - 115 | 3 |
| NC68 | TS | 4 | 29 | 7.3 | per 10 min | 2.6 | 1 - 15 | 3 |
| NCX1 | TS | 18 | 0 | 0.0 | per 10 min | 0.0 | 0 - 0 | 1 |
| NCX2 | TS | 4 | 0 | 0.0 | per 10 min | 0.0 | 0 - 0 | 1 |
| NCX3 | TS | 2 | 0 | 0.0 | per 10 min | 0.0 | 0 - 0 | 1 |
| NCX4 | TS | 4 | 0 | 0.0 | per 10 min | 0.0 | 0 - 0 | 1 |
| NCX5 | TS | 8 | 0 | 0.0 | per 10 min | 0.0 | 0 - 0 | 1 |
| NCX6 | TS | 6 | 0 | 0.0 | per 10 min | 0.0 | 0 - 0 | 1 |
| NCX7 | TS | 15 | 0 | 0.0 | per 10 min | 0.0 | 0 - 0 | 1 |
| VAN52b | TS | 12 | 18 | 1.5 | per 10 min | 0.7 | 0 - 8 | 2 |
| VAN53 | TS | 126 | 131 | 1.0 | per 10 min | 0.1 | 0 - 11 | 2 |
| VAN54 | TS | 75 | 22 | 0.3 | per 10 min | 0.1 | 0 - 4 | 1 |
| VAN55 | TS | 55 | 61 | 1.1 | per 10 min | 0.3 | 0 - 11 | 2 |
| VAN56 | TS | 45 | 2 | 0.0 | per 10 min | 0.0 | 0 - 1 | 1 |
| VAN10 | TR | 98 | 31 | 31.6 | per ha | 5.7 | 0 - 200 | 2 |
| VAN10b | TR | 219 | 319 | 145.7 | per ha | 17.5 | 0 - 1800 | 3 |
| VAN10c | TR | 90 | 4 | 4.4 | per ha | 2.2 | 0 - 100 | 1 |
| VAN16 | TR | 271 | 1958 | 722.5 | per ha | 60.0 | 0 - 7000 | 3 |
| VAN17 | TR | 88 | 490 | 556.8 | per ha | 130.7 | 0 - 7000 | 3 |
| VAN18 | TR | 258 | 712 | 92.0 | per ha | 13.4 | 0 - 2633 | 3 |
| VAN21 | TR | 215 | 76 | 35.3 | per ha | 6.6 | 0 - 800 | 3 |
| VAN26 | TR | 102 | 284 | 139.2 | per ha | 44.3 | 0 - 4050 | 3 |
| VAN5 | TR | 89 | 160 | 179.8 | per ha | 28.5 | 0 - 1500 | 3 |
| VAN5b | TR | 86 | 58 | 67.4 | per ha | 12.5 | 0 - 500 | 3 |
| VAN52 | TR | 40 | 24 | 60.0 | per ha | 13.8 | 0 - 300 | 3 |
